# Harnessing the power of tumor-draining lymph nodes: unveiling predictive biomarkers for immune checkpoint inhibitor

**DOI:** 10.37349/etat.2025.1002315

**Published:** 2025-05-13

**Authors:** Zihan Chen, Jiangnan Yu, Zhikun Guo, Shuxian Chen, Yina Li, Qian Zhou, Lei Wang

**Affiliations:** IRCCS Istituto Romagnolo per lo Studio dei Tumori (IRST) “Dino Amadori”, Italy; International Cancer Center, Shenzhen University Medical School, Shenzhen 518054, Guang Dong, China

**Keywords:** Tumor-draining lymph nodes, immune checkpoint inhibitors, cancer-immunity cycle, predictive biomarker

## Abstract

With the escalating application of immune checkpoint inhibitors (ICIs) in solid tumors, these therapies have demonstrated clinical benefits but remain hampered by relatively low response rates. Reliable biomarkers to predict ICIs responsiveness are essential for selecting appropriate patients and optimizing therapeutic outcomes. Given the pivotal role of tumor-draining lymph nodes (TDLNs) in orchestrating systemic antitumor immunity, their intrinsic features—such as dynamic organization in T cell subsets and functional status of antigen-presenting cells, hold considerable potential as predictive biomarkers for ICIs. Moreover, the complexity of ICIs-induced responses in TDLNs necessitates integrating multiple biomarkers for accurate prediction. Through continuous refinement of predictive strategies, TDLNs are poised to play an indispensable role in enhancing ICIs efficacy and guiding personalized immunotherapy. Here, we provide a review to discuss the possibility of using the intrinsic features of TDLNs as a predictive marker for ICI therapy.

## Introduction

The advent of immune checkpoint inhibitors (ICIs) has revolutionized oncological therapy by restoring and enhancing anti-tumor immune responses in patients with solid tumors. ICIs achieve this by targeting inhibitory receptors and ligands on immune cells, such as programmed death protein 1 [PD-1, also named cluster of differentiation 279 (CD279)], programmed death ligand 1 (PD-L1, also named CD274), and cytotoxic T-lymphocyte associated protein 4 (CTLA-4, also named CD152), thereby blocking their interactions with ligands or receptors then alleviating tumor-induced immunosuppression [1]. In recent years, ICIs have demonstrated significant clinical benefits in treating specific malignant tumors, including melanoma, non-small cell lung cancer (NSCLC), colorectal cancer, renal cell carcinoma (RCC), and triple negative breast cancer (TNBC) [2–7]. However, the overall patient response rate to anti-PD-1/PD-L1 and anti-CTLA-4 monoclonal antibody therapies remains suboptimal in clinical practice [1, 7–11], with a maximum of 30% to 50% of patients responding to PD-1 blockade [12, 13]. This reality underscores the urgent need for developing precision predictive biomarkers.

Predictive biomarkers refer to objective indicators that prospectively evaluate the effectiveness of a patient’s response to specific treatments, identifying populations more likely to benefit (or suffer adverse effects) from particular interventions, thereby enabling precision medicine. An ideal predictive biomarker should possess the following characteristics: 1) high specificity and sensitivity to accurately distinguish responders from non-responders, minimizing false positives and negatives; 2) strong reproducibility and stability; 3) clinical utility, with standardized detection methods, cost-effectiveness, and ease of implementation in clinical practice. Biomarker detection methods are diverse, including antibody-based assays [immunohistochemistry, enzyme-linked immunosorbent assay (ELISA)], mass spectrometry for metabolite or proteome quantification, sequencing-based approaches [polymerase chain reaction (PCR), next-generation sequencing for gene mutations], and cell-based assays (flow cytometry for immune cell surface markers) [14].

Current predictive biomarkers for ICI efficacy encounter limitations. First, the clinical gold standard—quantitative analysis of PD-L1 expression in tumor tissue is constrained by tumor heterogeneity [15], post-translational modifications [16], and inconsistencies in detection antibodies and platforms [17]. Moreover, clinical data shows that PD-L1-negative patients may still benefit from PD-1 inhibitors [18]. Second, other tissue-based biomarkers, such as high tumor mutational burden (TMB) and microsatellite instability (MSI), exhibit high specificity but apply to only a small fraction of patients [19, 20]. Tumor major histocompatibility complex class I (MHC-I) expression, another key predictive marker, is dynamically regulated by factors like interferon-γ (IFN-γ) signaling, epigenetic modifications, and tumor microenvironmental stress [21]. Even with high MHC-I expression, tumors can evade immune destruction through mechanisms such as PD-L1 upregulation, regulatory T cells (T_reg_) infiltration, or antigen presentation defects [22]. Importantly, these tumor-centric biomarkers fail to fully reflect systemic immune status, driving our focus toward tumor-draining lymph nodes (TDLNs) as critical immune regulatory hubs.

As pivotal nodes in the tumor immune cycle, TDLNs reflect systemic immune activation states and therapeutic responses to ICIs. The unique architecture of TDLNs (e.g., paracortical zones and lymphoid follicles) provides an ideal microenvironment for interactions among T cells, B cells, and antigen-presenting cells (APCs). Variations in immune cell subsets, metabolic dynamics, and other features within TDLNs collectively form a multidimensional index system for assessing ICI efficacy. This review innovatively integrates TDLN characteristics into the framework of tumor immunity theory, providing fresh insights into predicting ICI outcomes. Mining predictive biomarkers from TDLNs, which serve as the “training hubs” for systemic immunity, will break through the traditional tumor-centered limitations and possess great clinical potential in precision immunotherapy.

## ICIs efficacy requires mobilization of the systemic immune cycle

### Immune checkpoints and ICIs

T cell activation mainly requires two signals: the first signal is generated by the binding of MHC-presented antigens to the T cell receptor (TCR), while the second signal involves co-stimulatory and co-inhibitory signaling pathway. However, T cells are not continuously activated, in order to prevent the immune system from over-activating and damaging its own tissues. This regulation is achieved through a series of signaling molecules on the surface of activated T cells, which act as “brakes” on T cell activation [1, 23]. These negative regulatory signaling molecules are known as immune checkpoints, which play a crucial role in maintaining immune homeostasis.

Typical immune checkpoint molecules include PD-1, PD-L1, and CTLA-4. PD-1 is expressed on activated T cells and in non-lymphoid tissues, and thus plays a major role in maintaining peripheral tolerance [24, 25]. Those molecules binding with their ligands inhibit TCR-mediated T cell proliferation and cytokine secretion [26]. PD-L1 is a ligand for PD-1 expressed by tumor cells, stromal cells, and immune cells such as lymphocytes and myeloid cells. The PD-1/PD-L1 pathway regulates immune tolerance within the tumor microenvironment (TME). CTLA-4 is induced upon T cell activation and primarily modulates T cell responses mainly in the early stages within lymphoid organs. For competing with co-stimulatory molecule CD28 for binding to B7 proteins, CTLA-4 prevents excessive immune responses, thereby protecting normal tissues and avoiding autoimmunity [26].

However, many tumor cells also “exhaust” T cells by overexpressing checkpoint molecules that bind to T cell co-inhibitory receptors, thereby limiting the body’s anti-tumor immune response and promoting immune escape. Based on this mechanism, ICIs are developed to block the binding of immune checkpoints to their ligands, relieving the immune suppression of T cells and reactivating the immune cells to exert anti-tumor effects, thus enhancing the body’s anti-tumor immune response [1]. Typical ICIs include monoclonal antibodies against PD-1, PD-L1, and CTLA-4, which have been approved for clinical use in treating various solid tumors such as melanoma [3, 27, 28], NSCLC [2, 29–31], RCC [5, 13, 30, 32], and TNBC [4, 33–35].

### Cancer-immunity cycle

It is important to point out that it was previously believed that the immune response induced by ICIs mainly occurs within the tumor, particularly in the TME [36, 37]. However, a growing number of papers suggests that ICI-induced immune responses occur not only in the TME, often referred to as the “battlefield”, but also highlight the essential role of various cell types in TDLNs, which serve as the “training camp” for immune cells [38–41].

**Figure 1:**
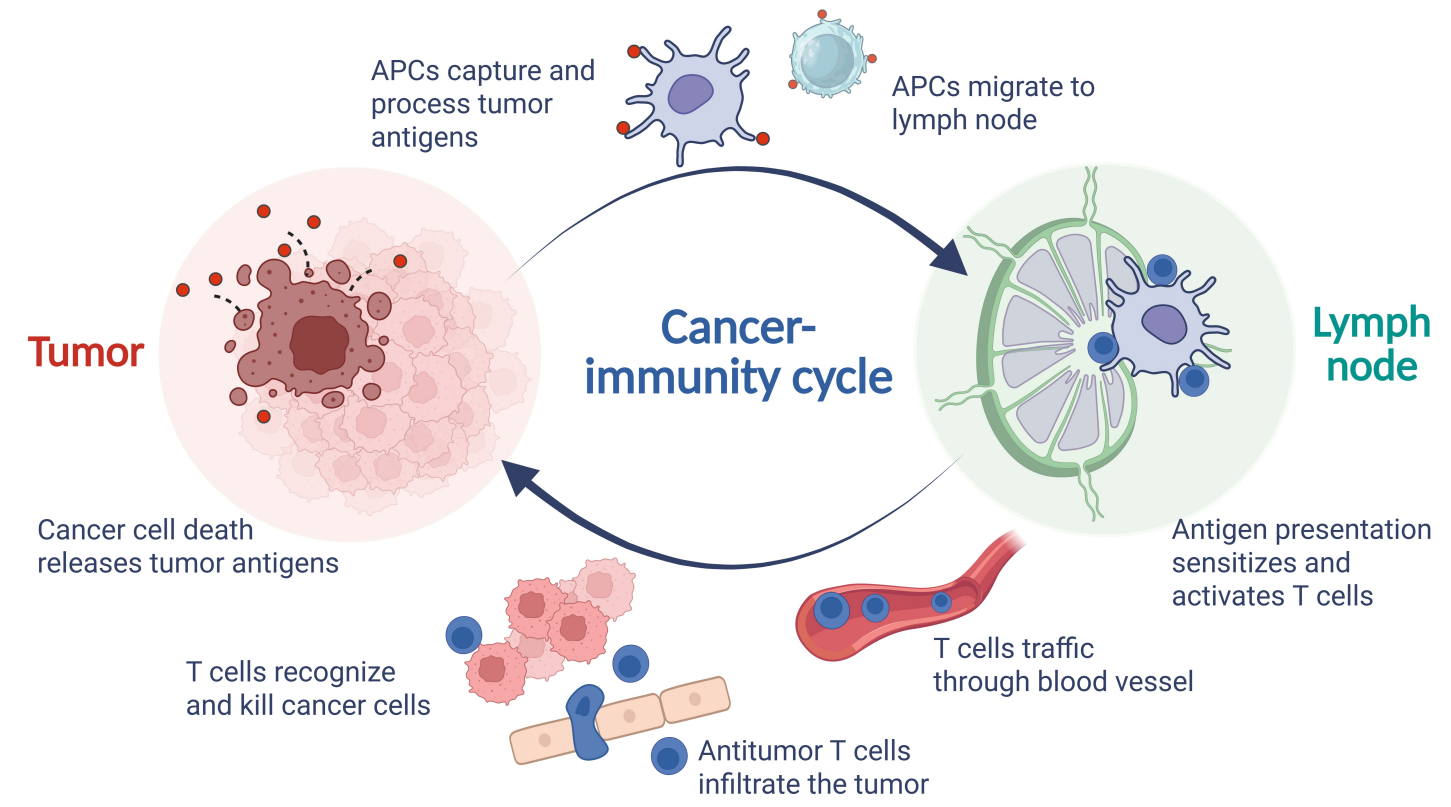
**The process of cancer-immune cycle.** APCs: antigen-presenting cells. Created in BioRender. Wang, L. (2025) https://BioRender.com/z43p804

According to the cancer-immunity cycle, tumor antigens, which include tumor-associated antigens (TAAs) and tumor-specific antigens (TSAs), released from dying tumor cell death following immunotherapy are captured by APCs. These APCs then present immunogenic tumor antigens to naive T cells (T_naive_) in the lymph nodes and make decisions about tumor tolerance or immune activation [42]. Activated effector T cells (T_eff_) leave the lymph node and infiltrate the tumor, specifically recognizing and killing cancer cells, thereby releasing additional tumor antigens. These TAAs/TSAs released during tumor cell death, are subsequently captured by APCs in the TME, perpetuating the immune cycle. Figure 1 illustrates a brief process of the cancer-immune cycle.

The efficacy of ICIs hinges on the mobilization of a systemic immune cycle, a coordinated series of immune responses involving both the tumor TME and peripheral immune organs especially in the TDLNs, facilitating the activation, proliferation, and infiltration of T_eff_ that sustain tumor cell destruction. Therefore, TDLNs have the potential to serve as valuable “windows of observation” for predicting ICIs efficacy.

## TDLNs as critical sites for generating antitumor immunity

Although the presence of tumor cells in the TDLNs is a negative prognostic factor in many tumor types, controversy remains regarding the therapeutic benefit of immediate removal of the remaining lymph nodes. In solid tumor types such as melanoma, thyroid cancer, and breast cancer, prophylactic lymph nodes removal has not been shown to improve patient survival [43, 44].

As key hubs of tumor immune response, TDLNs serve dual roles: they act as outposts for tumor metastasis, and as essential sites for tumor antigen presentation and anti-tumor immune cell activation. Due to this dual functionality, the potential value of TDLNs in predicting the response to ICIs has garnered increasing attention [1]. More importantly, previous studies have shown that the status of TDLN immune cell subsets, cytokines types, and metabolites [e.g., indoleamine 2,3-dioxygenase 1 (IDO1) overexpress] levels are closely related to the efficacy of ICIs [38, 39, 45, 46], suggesting the potential role of intrinsic characteristics of TDLNs as biomarkers for ICIs therapy.

### TDLNs anatomy

The lymph node, a highly organized immune organ, is situated at the confluence of the lymphatic system’s blood vessels and can be anatomically subdivided into three primary regions: the B cell zones, T cell zones, and medulla zones [47]. Each zone plays a distinct role in the node’s immune functions.

#### T cell zones (paracortical region)

**Figure 2:**
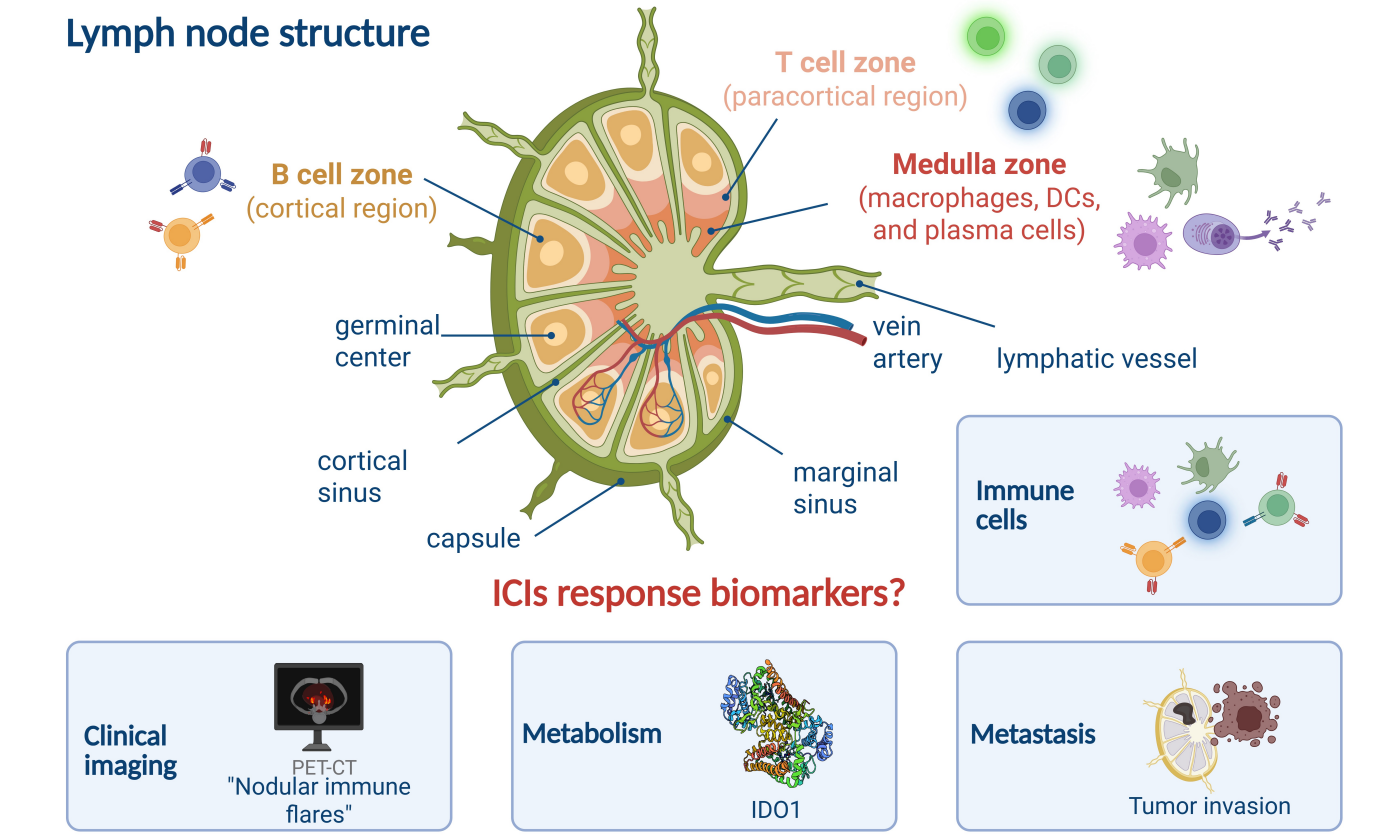
**Lymph node structure and major features of ICIs response.** DCs: dendritic cells; ICIs: immune checkpoint inhibitors; IDO1: indoleamine 2,3-dioxygenase 1; PET-CT: positron-emission tomography-computed tomography. Created in BioRender. Wang, L. (2025) https://BioRender.com/q07x744

The T cell zone, also known as the paracortical zone, is located in the deeper layers of the cortical region. This area is densely populated with T cells, particularly CD4⁺ and CD8⁺ T cells, which are crucial for cell-mediated immunity. The paracortical zone serves as a hub for T cell activation, proliferation, and differentiation, facilitating immune responses against foreign antigens. Dendritic cells (DCs) migrate to lymph nodes via CCL19/CCL21 chemokine signaling after capturing peripheral antigens. Through MHC molecules presenting antigenic peptides, they form immunological synapses with T_naive_, thereby activating both CD4⁺ T helper (T_H_) cells and CD8⁺ CTLs. Additionally, fibroblastic reticular cells (FRCs) in the paracortical region support T cell survival and maintain lymph node T cell zones by secreting cytokine interleukin-7 (IL-7) and constructing three-dimensional reticular scaffolds. Figure 2 presents the compartmental structure of the lymph node, the composition of its internal immune cells, and major features as biomarkers.

#### B cell zones (cortical region)

Beneath the outer peritoneum lies the cortical region, which is the B cell zone. This area is repleted with lymphoid follicles primarily composed of B cells. Among them, Primary lymphoid follicles are primarily composed of quiescent B cell clusters. Following antigen stimulation, germinal centers (GCs) form within these follicles, transforming them into secondary follicles. B cell zone is the center of B cell activation and differentiation, where B cells mature and undergo class-switch recombination to produce antibodies against specific antigens.

#### Medulla zones

The medulla zone is positioned in the central region of the lymph node and is home to plasma cells which would secret antibody and antigen presentation, clearance cells like DCs and macrophages. The medulla zone also contains high endothelial venules (HEVs) that facilitate the entry of naive lymphocytes from the bloodstream and the exit of lymphocytes back into the lymphatic circulation via efferent lymphatic vessels.

Together, these distinct zones within the lymph node collaborate to form a cohesive and robust immune defense system, capable of recognizing and eliminating foreign antigens and pathogens.

### TDLNs required for ICIs treatment response

Removal of lymph nodes, especially TDLNs, has been found to negatively impact ICIs therapeutic efficacy in mouse models. Chamoto et al. [48] demonstrated that deleting of CD8^+^ T cells or ablating of TDLNs in PD-L1 mAb-treated mouse model of colon cancer resulted in impaired tumor growth inhibition by the PD-L1 mAb, suggesting TDLNs play a critical role in the initiation of CD8⁺ T cells and the generation of CTLs during PD-1 blockade therapy. Similarly, Fransen et al. [39] highlighted the significant involvement of TDLNs in the therapeutic efficacy of PD-1 and PD-L1 inhibitors in the MC38 colon cancer mouse model. Immune activation induced by ICIs was primarily observed in TDLNs rather than non-draining lymph nodes, characterized by the local accumulation of CD8^+^ T cells. These studies underscore the importance of TDLNs as sites where APCs interact with T cells to initiate tumor-specific CTL amplification. Given that many ICIs target this interaction between tumor and immune cells, the microenvironment within the TDLNs may significantly influence their efficacy.

## CD8^+^ T cell subsets in TDLNs

As critical hubs connecting primary tumors with systemic immune responses, TDLNs are increasingly recognized for the dynamic changes in lymphocytes within them as important biomarkers for evaluating antitumor immune effects and predicting the efficacy of ICIs [39]. In particular, T cell subsets within TDLNs, including CD4^+^ and CD8^+^ T cells, play pivotal roles in elucidating immune dynamics within TME and guiding clinical therapeutic decisions. T cells activated by DCs differentiate into T_eff_ within TDLNs, then migrate through lymphatic and blood vessels to infiltrate the TME where they recognize and eliminate tumor cells expressing cognate antigens. Additionally, a subset of CD8^+^ T cells in TDLNs differentiates into memory T cells (T_mem_), providing long-term immune protection.

### Stem-like CD8^+^ T cell

T cell immunity is an important part of the body’s immune defense against cancer. Upon antigenic stimulation, the T_naive_ differentiates into T_mem_ and T_eff_. T_mem_ serve as a functional reservoir for T_eff_, retaining the potential to re-differentiate into T_eff_ when needed. However, in anti-tumor processes, prolonged antigen exposure promotes terminal differentiation or “exhaustion” of T cells, which hinders the formation of T_mem_ [49]. These exhausted T cells exhibit sustained upregulation of inhibitory receptors (including PD-1, LAG-3, TIM-3, CTLA-4, etc.), which suppress the TCR signaling pathway through SHP-1/2 phosphatases. This leads to reduced secretion of IFN-γ and tumor necrosis factor-α (TNF-α), ultimately resulting in the loss of cytotoxic T cell tumor-killing capacity and consequently diminishing the efficacy of ICIs.

Notably, there exists a unique subset of CD8^+^ T cells that possess stem-like characteristics, including self-renewal and multilineage differentiation capabilities, encompassing both stem-like exhausted T cells and T_mem_. These cells play a pivotal role in antitumor immunity, particularly in the context of ICIs therapy. They are among the cellular populations capable of effectively responding to treatment and mediating sustained antitumor immune responses [50]. Typically, these stem-like CD8⁺ T cells express high levels of the transcription factor T cell factor 1 (TCF1)/TCF7 while maintaining low levels of effector differentiation markers [51]. With higher proliferative potential and stem cell-like developmental plasticity, these stem-like CD8^+^ T cells not only survive for a longer period of time under conditions of sustained antigenic stimulation, but also regulate immune responses by differentiating into effector or depleted T cells [51, 52].

Stem-like CD8^+^ T cells have been found to differentiate into two major classes: CD8^+^ precursor exhausted T cells (T_pex_), which maintain the immune function of specific CD8^+^ T cells in a chronic antigen-stimulated environment and are considered to be important target cells for PD-1/PD-L1 blockade therapy [53]; and tumor-specific T_mem_, which are usually generated in TDLNs and act as key responders of anti-tumor immunity in ICIs therapy [54].

#### Progenitor exhausted CD8^+^ T cells

A subset of T_eff_ differentiates into TCF1-expressing “stem-like” CD8^+^ T cells, referred to as T_pex_. Due to their self-renewal and high proliferative capacity, T_pex_ are considered a critical population for sustaining specific CD8^+^ T cell responses under chronic antigenic stimulation [49, 55]. The presence and activity of T_pex_ are closely associated with the ICIs therapeutic efficacy. During ICIs therapy, T_pex_ predominantly expand and differentiate into terminally exhausted T cells (T_ex-term_), thereby increasing the population of T_ex-term_ within the TME and enhancing anti-tumor immune responses [51, 52].

In 2021, Connolly et al. [56] identified a population of TCF1-expressing “stem-like” CD8^+^ T cells within TDLNs, referred to as T_pex_. Miller et al. [52] demonstrated that T_pex_ mediate tumor control and respond to checkpoint blockade to varying degrees. Among these, T_pex_ exhibited a greater capacity for tumor control compared to T_ex-term_, and was identified as the primary subset responding to PD-1/PD-L1 blockade. Another study in human head and neck squamous cell carcinoma (HNSCC) found that T_pex_ were abundantly present in uninvolved regional lymph nodes (uiLNs) and clonally related to T_ex-term_ in tumors [57]. After anti-PD-L1 immunotherapy, T_pex_ in uiLNs were activated and underwent differentiation, as evidenced by the presence of activated T_pex_ and transitional exhausted T cells (T_ex-int_) localized near DCs. An increase in T_ex-int_ was observed in both uiLNs and peripheral blood. However, in lymph nodes with established tumor metastases, the responsiveness of T_pex_ and T_ex-int_ to anti-PD-L1 therapy was impaired [57].

These findings suggest that T_pex_ can be activated during ICIs therapy and play a key role in anti-tumor immune responses. The consistent activation and differentiation of T_pex_ in uiLNs, along with their mobilization into circulation following anti-PD-L1 treatment, indicate effective functioning of the body’s immune microenvironment. Furthermore, the robust responsiveness of T_pex_ in tumor-free TDLNs to anti-PD-L1 therapy underscores their potential as predictive biomarkers for ICI treatment outcomes.

#### Tumor-specific T_mem_ in TDLNs

Following the discovery that progenitor exhausted T cells functionally respond to PD-1 ICIs, researchers further identified another population of stem-like CD8^+^ T cells: tumor-draining lymph node derived tumor specific T_mem_ (TdLN-TTSM) [54]. Unlike T_pex_, TdLN-TTSM cells do not express the depletion marker thymocyte selection associated high mobility group box (TOX) and have low levels of PD-1 expression. These cells are phenotypically similar to central T_mem_, with high expression of memory-associated markers such as CD122, CD127, and CD62L. Additionally, TdLN-TTSM cells are identified as the main responders to PD-L1 ICIs therapy. These cells can expand and differentiate within the TME independently of persistent antigen stimulation, underscoring their pivotal role in sustaining long-term antitumor immunity.

Functionally, upon re-encountering tumor antigens, TdLN-TTSM cells are able to rapidly proliferate and differentiate into T_eff_, which produce a large number of cytokines, such as IFN-γ and TNF-α, to effectively kill tumor cells or control tumor growth and exhibit remarkable efficacy in ICIs therapy in mouse tumor model.

Thus, monitoring the quantity and functionality of TdLN-TTSM cells may help predict patient responsiveness to ICIs therapy, providing valuable guidance for personalized treatment strategies. Furthermore, exploring the role of TDLNs and related T_pex_ in other tumor types could enhance the utility of predictive biomarkers, facilitating the broader application of cancer immunotherapy.

### CD28^+^ CD8^+^ T cells

As mentioned previously, two signals are required for T cell activation in the body: a primary antigen-inducing signal from the TCR and a secondary signal from a co-stimulatory receptor [47]. CD28 is precisely a key T cell co-stimulatory molecule and member of the immunoglobulin superfamily, which is mainly expressed on the surface of T cells, especially on initial T cells and T_mem_. It provides the co-stimulatory signals required for T cell activation and proliferation by binding to B7-1 (CD80) and B7-2 (CD86) on APCs, thereby lowering the activation threshold of the TCR complex and promoting T cell survival and effector functions [47, 58]. As a critical co-stimulatory molecule for T cell activation and survival, CD28 is expressed on all T_naive_ in neonates. However, as T cells undergo prolonged antigen stimulation and differentiation, particularly during chronic viral infections and within the TME, they may lose CD28 expression [59]. This phenotypic change is associated with T cell exhaustion, characterized by high levels of inhibitory receptors such as PD-1 and impaired functionality, with the absence of CD28 exacerbating this process [60]. Furthermore, the co-stimulatory signaling function of CD28 can be competitively inhibited by ligands such as CTLA-4. Due to its structural homology with CD28 and higher binding affinity, CTLA-4 can outcompete CD28 for ligands, thereby suppressing T_eff_ responses [47]. Consequently, CD28 expression on T cells is not only a key biomarker for assessing T cell exhaustion but also a homologous target of the CTLA-4 immune checkpoint, and its presence is associated with ICIs treatment response.

Kamphorst et al. [61] demonstrated in both mouse models and samples from patients with advanced NSCLC that PD-1-targeted therapy enhances T cell responses, but this rescue requires the involvement of the CD28/B7 co-stimulatory pathway. In mouse models of colorectal cancer and melanoma, they found that the CD28/B7 co-stimulatory pathway is essential for effective PD-1 therapy during tumor burden and chronic viral infections. Conditional gene deletion studies showed that the proliferation of CD8^+^ T cells after PD-1 blockade depends on the presence of CD28, and B7 co-stimulation is also necessary for effective PD-1 therapy. Additionally, their group analyzed blood samples from advanced NSCLC patients and observed that the proliferating CD8^+^ T cells following PD-1 therapy were predominantly CD28-positive. Specifically, in approximately half of the patients, an increase in Ki-67-expressing CD8^+^ T cells was observed after PD-1 treatment, primarily within the PD-1^+^ population, and these PD-1^+^ CD8^+^ T cells were mainly CD28^+^. This suggests that PD-1 therapy induces the selective proliferation of CD8^+^ T cells, highlighting the potential of CD28 as a biomarker for predicting CD8^+^ T cell responses in cancer patients.

Further supporting the importance of CD8^+^ T cells, studies by Duraiswamy et al. [62] found that tumor-specific CD8^+^ T cells accumulate in ovarian cancer niches and are closely associated with intratumoral myeloid APCs. These APCs support tumor-infiltrating T cells and provide CD28 co-stimulation during PD-1 blockade therapy, enabling sustained antitumor immune responses and facilitating the efficacy of PD-1 blockade.

Collectively, these findings underscore the necessity of CD28 co-stimulation for rescuing CD8^+^ T cells [38], highlight the critical role of the CD28/B7 pathway in PD-1 therapy for cancer patients, and suggest that increased expression of CD28 on PD-1^+^ CD8^+^ T cells may indicate a better response to ICIs.

## CD4^+^ T cell subsets in TDLNs

CD4^+^ T cells possess a wide range of functions that are dependent on specialization through functional polarization. Upon recognition of MHC-II complexes presented by APCs, the initial CD4^+^ T cells are initiated and undergo clonal expansion and differentiation [63]. Depending on a variety of factors such as the cytokine environment of the microenvironment, the intensity of the TCR stimulation, and the type and functional state of the APCs involved, the initial CD4^+^ T cells ultimately form distinct CD4^+^ T_H_ subsets [64]. In addition to differentiating into distinct CD4^+^ T cell subpopulations, CD4^+^ T cells may also exhibit environment-dependent effector activity [65]. CD4^+^ T-rich subpopulations play diverse functions in antitumor immunity. In TDLNs, extremely large numbers of T_naive_ scan specialized APC-presented TAAs, in which different types of CD4^+^ T cells support the antitumor immune response, which is not only an adjuvant role of CD4^+^ T cell subsets to CD8^+^ T cell, but also acts as an antitumor agent by directly attacking tumor cells and their stromal support. It has been found that successful activation of TAA-specific CD4^+^ T cells is often critical for anti-tumor effects in vivo, and that the T_H_1 CD4^+^ T cells-derived effector cytokines IFN-γ and TNF can influence tumor progression by directly targeting either tumor cells or stromal compartments within the tumor tissue [63]. However, T_reg_, a typical CD4^+^ T cell subset, have also been found to support tumor progression and promote tumor persistence and metastasis.

### T_H_1 CD4^+^ T cells

CD4^+^ T cells exhibiting an activated T_H_1 phenotype serve as biomarkers that predict response to ICIs therapy. A study showed that the combination of anti-PD-L1 and anti-CTLA-L1 in patients with recurrent or metastatic HNSCC, compared to anti-PD-L1 therapy alone, is more likely to affect tumor progression [66]. The combined therapy effectively activates and expands T_H_1-type CD4^+^ cells, which correlated with a favorable response to the combination immunotherapy. The article highlights that pre-existing T_H_1 immunity predicts expansion following anti-PD-L1^+^ anti-CTLA-4 treatment. Researchers compared changes in T cells within TDLNs and tumor tissues of patients treated with the combined therapy, and found that TDLNs consisted mainly of initial/central memory CD4^+^ T cells, whereas tumors were dominated by T_eff_. Analysis of TCR-seq data from tumors and TDLNs revealed that under dual treatment with anti-PD-L1 and anti-CTLA-4, initial/central memory CD4^+^ T cells at the site of the TDLNs were recruited, and differentiated and expanded under anti-CTLA-4 treatment, thereby transforming into T_eff_ to achieve tumor cell killing. This indicates that TDLNs are important sites for immune antigen depots and anti-tumor T cell training. Thus, CD4^+^ T cell activation and recruitment from TDLNs are hallmarks of early response to anti-PD-L1 plus anti-CTLA-4 in HNSCC.

In addition, researchers conducted a detailed analysis of patient tumor tissue sections. Spatial single-cell proteomic results showed that expanded CD4^+^ T cells and CD8^+^ T cells exhibited more pronounced co-localized in the tumor tissues of patients receiving combination therapy. Moreover, the expansion of these T cell populations was closely associated with the co-localization of DCs and plasma cells. This suggests that a similar spatial analysis of the lymph nodes could identify immune cells that contribute to the phenotypic changes in how such CD4 T cells become T_H_1-type, such as IL-12 production by DCs and macrophages, and IFN-γ production by natural killer (NK) cells and CD4^+^ T_H_1 cells themselves, which is essential for CD4^+^ T_H_1 cell differentiation. Therefore, it can be assumed that activated CD4^+^ T_H_1 cells are not only a biomarker of ICI treatment response, but also a key predictor of the efficacy of combination immunotherapy.

Recently Nature also reported the existence of a stem-like CD4^+^ T cells within TDLNs that are capable of differentiating into CD4^+^ T_H_1 cells and promoting effector differentiation of CD8^+^ T cells by secreting IFN-γ, thereby controlling tumor growth and improving response to ICI therapy [67]. This is the first identification of a population of PD1^+^ TCF1^+^ CD4 T cells that reside predominantly in TDLNs and exhibit the ability to self-renew and differentiate into classical CD4^+^ T_eff_, and are therefore considered stemness. In the context of T_reg_ cell depletion, these stem-like CD4^+^ T cells can differentiate into CD4^+^ T_H_1 cells and are augmented by the production of IFN-γ to drive the differentiation of stem-like CD8^+^ T cells into effector cytotoxic T cells. Notably, patients with RCC with a strong CD4^+^ T_H_1 response have been associated with a higher frequency of cytotoxic effector CD8^+^ T cells after ICI treatment and an improved progression-free survival. Thus, differentiation of PD1^+^ stem-like CD4^+^ T cell cells into CD4^+^ T_H_1 cells is not only critical for orchestrating effective antitumor immunity, but also predicts patients who are more likely to respond well to ICIs therapy.

### CD4^+^ T_reg_

T_reg_ are a heterogeneous population of CD4^+^ T lymphocytes with immunosuppressive functions, which play a key role in maintaining immune tolerance, preventing autoimmune diseases, and modulating immune responses. T_reg_ exert immunosuppressive functions through multiple mechanisms, such as secreting immunosuppressive cytokines [e.g., IL-10, transforming growth factor-β (TGF-β)], depleting key growth factors such as IL-2, and generating immunosuppressive cytokines through cell contact, as well as inhibiting the activation and proliferation of T_eff_ via cell contact. In tumor immunity, T_reg_ are regarded as key participants in immune escape within the TMEs and TDLNs, providing support for tumor immune privilege by suppressing anti-tumor effector immune responses. This makes them potential negative predictive markers in ICIs therapy.

TDLNs serve as the primary site for the induction and activation of de novo tumor-specific T_reg_, which maintain the immune tolerance within the TDLNs by inhibiting the function of T_eff_. This phenomenon mirrors the immunosuppression observed in the TME, allowing sustained tumor growth despite high levels of circulating tumor-specific T cells [68]. Huang et al. [69] found that T_reg_ in the TDLNs secreted TGF-β, which significantly inhibited the cytotoxic activity of tumor-specific CD8^+^ T cells, thereby promoting tumor growth in mouse tumor models. The presence of T_reg_ also affects the differentiation of CD4^+^ T cells. Alonso et al. [66] reported in a study of lung adenocarcinoma mouse model that CD4^+^ T cell differentiation in TDLNs favors the generation of T_reg_ over effector CD4^+^ T cells. In 2024, Cardenas et al. [67] published an article in Nature that showed that the differentiation of stem-like CD4^+^ T cells in TDLNs was a major factor in the growth of tumor cells, with T_reg_ in TDLNs being more prone to producing TGF-β compared to effector CD4^+^ T cells.

In addition to inhibiting CD8^+^ T cell function or affecting tumor antigen-specific CD4^+^ T cell differentiation, T_reg_ also impact the overall function of the cancer-immunity cycle. Through surface expression of CD39 and CD73, T_reg_ catalyze the degradation of ATP into adenosine, which subsequently downregulates the expression of adhesion molecules (such as ICAM-1 and VCAM-1) on tumor vascular endothelial cells via A2A adenosine receptor signaling, thereby reducing immune cell infiltration into tumors [70]. Additionally, peripheral blood T_reg_ can suppress systemic antitumor immunity through both adenosine-dependent and independent mechanisms, and their abundance is negatively correlated with the efficacy of ICIs [71].

Besides, T_reg_ can shape the immune-tolerant environment of lymph nodes by modulating PD-L1 expression and thus affect ICIs efficacy. A mouse model of lymph node metastasis melanoma constructed by Reticker-Flynn et al. [72] revealed a critical role for lymph nodes in tumor immune escape and distant metastasis. The lymph nodes formed an immunosuppressive microenvironment by upregulating the expression of MHC-I and PD-L1, which not only facilitated tumor cells’ evasion from NK cell mediated killing, but also suppressed T cell activity. This suppression generated tumor-specific immune tolerance, creating favorable conditions for distant tumor colonization and growth. The “Metastatic Tolerance” model proposed in the article emphasizes that lymph node metastasis not merely serves as a transit point for tumor cells to spread to distant sites, but rather induces tumor-specific immune tolerance, making distant tissues more receptive to tumor cell colonization. In this process, T_reg_ play a key role. Besides secreting immunosuppressive cytokines such as TGF-β, T_reg_ are closely associated with PD-L1 expression. Despite the possibility of a false negative, PD-L1 expression remains an important predictor of response to ICIs therapy, it can be hypothesized that T_reg_ within lymph nodes and the immune tolerance microenvironment they induce play a crucial role in predicting and modulating the response to ICIs therapy.

## B cell and antibody production in TDLNs

During tumor immunity, tumor-infiltrating B cells present antigens to MHC-II molecules via B cell receptors (BCRs) on their surface to promote T cell recognition of TAAs, and tumor-infiltrating plasma cells also produce a large number of cytokines and antibodies that are directly involved in the antitumor immune response [73]. These antibodies can promote anti-tumor immunity by driving antibody-dependent cellular cytotoxicity (ADCC) and phagocytosis, complement activation, and enhancement of antigen presentation by DCs [74, 75]. A study showed that dynamic changes in the BCRs pool may serve as a biomarker for predicting the clinical response to immunotherapy, and that the combination of anti-PD-1 and Toll-like receptor 9 (TLR9) agonists significantly increased the number of B cells in lymph nodes and the diversity of clonotypes of BCRs in a mouse model of colon and lung cancers, and that a reduction in the clonogenicity of the BCRs was negatively correlated with the effect of tumor suppression. In addition, this study found that combination therapy was able to increase tumor-specific antibodies secreted by B cells and trigger Fc-dependent tumor lysis [76].

Furthermore, in the TME, B cells are involved in the formation of tertiary lymphoid structures (TLSs), TLSs refer to acquired immune cell aggregates with well-defined tissue architectures that form ectopically in non-lymphoid tissues under non-physiological conditions. They are recognized as hallmarks of immune cell infiltration into solid tumors. Consequently, the presence of such B cells is often associated with a good response to ICIs treatment [77, 78], possibly because B cells instruct T cells (especially CD8^+^ T cells) to recognize TAAs and enhance local immune responses [79]. Further studies are needed to investigate whether such B cell subpopulations in TDLNs would also have the same function as B cells in TLS. However, it can be speculated from the role played by B cells within the TLS in the TME that B cell responses within the TDLNs may be of therapeutic and predictive value in improving ICIs therapy.

Compared with T cells, the study of immune checkpoints of B cells in tumors is still in its infancy [80], but it has been found that many immune checkpoints on the surface of B cells in the TME are upregulated, such as CTLA-4 [81], TIGIT [82], PD-L1 [83], TIM-1 [84], etc., which affects the functions of B cells, such as proliferation, differentiation, antigen presentation, and antibody production [85], and renders the anti-tumor effects of B cells ineffective. Meanwhile, abnormal B cell immune checkpoint signaling not only affects the function of B cells themselves, but also regulates the tumor-killing effect of other immune cells, promotes the formation of immunosuppressive TMEs and tumor immune escape, which may lead to the failure of immunotherapy. ICIs (e.g., PD-1/PD-L1 inhibitors) may, by blocking the immune checkpoint signaling of B cells, restore the proliferation, activation, antigen presentation, and co-stimulatory functions, thereby assisting T cells in exerting anti-tumor effects. For example, elevated proportions of plasma cells in the B-cell population, as well as an increase in memory B cells associated with tumor control [86], have been observed in NSCLC and RCC cohorts treated with PD-L1 antibodies [87]. These findings suggest that B-cell responses may have considerable therapeutic and predictive value for ICIs treatment, but further studies are needed to demonstrate whether there is a similar role specifically in TDLNs.

## Myeloid APCs in TDLNs

Myeloid APCs mainly including DCs and macrophages, are the central regulators of anti-tumor immune response. The trafficking and lymph node homing of APCs via blood and lymphatic vessels constitute the core engine driving the continuous operation of the cancer-immunity cycle. Specifically, APCs (particularly DCs) phagocytose apoptotic tumor cells, capture exosomes, or endocytose soluble antigens to acquire TSAs within the TME. Following antigen uptake, DCs upregulate CCR7 (which responds to the chemokines CCL19/21 in TDLNs), thereby initiating their migration toward TDLNs. Upon reaching TDLNs, APCs activate specific immune responses by capturing and processing antigens and presenting them in the form of MHC molecule-bound form to T cells [88]. ICIs therapy enhance the anti-tumor immune response by restoring T_eff_ activity through the blockade of immunosuppressive pathways, restoring the activity of T_eff_, thereby enhancing the anti-tumor immune response. In this process, the role of APCs in initial T cell activation is crucial, for instance, a study published in J Hepatol. (2022) identified distinct APCs and T_mem_ in the blood of HCC patients as biomarkers for distinguishing ICIs responders and immune-related adverse events (irAEs). Given the central role of lymph nodes as key sites where APCs and T cells interact, exploring these interactions within TDLNs holds even greater promise for understanding and optimizing the effects of ICIs therapy [89]. Besides, other studies have shown that enhancing DC function may improve and/or expand responsiveness to ICIs therapy [88]. Functional DCs may enhance immune surveillance of tumors by inducing potent CD8^+^ cytotoxic T cell responses. In addition, certain subtypes of DCs are strongly associated with the success of ICIs therapy [90, 91], which further promotes the activation of tumor-specific T cells through cross-presentation of antigens. Macrophages act as complementary APCs also play a role in tumor antigen presentation and immunomodulation [92], but the cells have also been shown to hinder antitumor immunity when subjected to the TME.

### DCs

DCs are able to acquire tumor antigens from the TME and activate CD8⁺ T cells and CD4⁺ T cells and maintain antitumor immune responses by cross-presentation via MHC-I molecules or direct antigen presentation via MHC-II molecules. As the most important dedicated APCs, functionally active DCs of different subtypes play an important role in the immunotherapeutic response, which is mainly related to the crosstalk or communication between DCs and antitumor T cells [93]. Preclinical data suggest that the antitumor effect of anti-PD-1 antibodies requires the interaction between DCs (especially cDC1) and T cells for “authorization”. Following PD-1 inhibition, activated CD8^+^ T cells secrete IFN-γ, which activates DCs, particularly cDC1, to produce IL-12. This cDC-secreted IL-12 further stimulates cytotoxic CD8^+^ T cells and enhances the recovery of T cell dysfunction mediated by anti-PD-(L)1 antibodies [92].

Studies have shown that the functional status of DCs is closely related to the efficacy of ICIs. For example, in human HNSCC patients treated with anti-PD-L1 showed a closer spatial localization of T_pex_ and DCs in uiLNs [57], which further validates the importance of DCs and antitumor T cells interactions in the treatment of ICIs; and in melanoma, activation of the β-catenin signaling pathway inhibits CD103⁺ DCs and T cells from entering the tumor, but by injecting bone marrow-derived DCs into the tumor site, T cell recruitment can be promoted, making the tumor more sensitive to PD-L1 and CTLA-4 monotherapy [94, 95]. Meanwhile, activation of TLR4 on CD103⁺ DCs enhances CD8⁺ T cell infiltration and significantly improves the efficacy of ICIs [95, 96]. The CCR7⁺ DC subpopulation also plays an important role in antitumor immunity, with mature DCs signaling via the chemokine CCR7 migrating from the TME to TDLNs, where they initially activate and expand tumor-specific T cells. This CCR7⁺ DC subpopulation exhibits high levels of PD-L1 expression and plays a key role in interaction with CD8⁺ T cells.

### Macrophages

Macrophages play multiple roles in the regulation of anti-tumor immune responses, with subpopulations that promote anti-tumor immunity and also exert immunosuppressive functions after being affected by the TME. Macrophages can be categorized into subcapsular sinus (SCS) macrophages and medullary sinus macrophages based on their anatomical location in the lymph node [97, 98]. Clinical studies have shown that the density of CD169^+^ SCS macrophages in TDLNs is significantly associated with a favorable patient prognosis. These macrophages function as APCs, activating T cell responses in lymph nodes and limiting the spread of tumor cells by capturing tumor-derived antigens or metastatic tumor cells [99]. In addition, CD169^+^ SCS macrophages are able to capture subcutaneously injected apoptotic tumor cells and activate CD8^+^ T cells through antigen presentation, thereby enhancing antitumor immunity.

In addition to acting as APCs, macrophages can also participate in the cytotoxicity and clearance of target cells through activating Fcγ receptors (FcγRI, FcγRIIA, FcγRIIC, and FcγRIIIA) on their surfaces, which are particularly prominent in monoclonal antibody therapy [95]. Studies have shown that ipilimumab, an antibody against CTLA-4, is able to reduce the number of T_reg_ through FcγR-mediated effects, thereby enhancing the activity of anti-tumor T cells [1, 100]. In addition, melanoma patients who responded to ipilimumab had a higher proportion of FcγRIIIA^+^ CD16^+^ monocytes, suggesting that activated Fcγ receptors may serve as a potential predictor of response to therapy.

On the other hand, macrophages may also limit antitumor immune responses in some cases, and tumor-associated macrophages are more likely to antagonize immune checkpoint therapy than tumor-infiltrating DCs. For example, the inhibitory Fcγ receptor (FcγRIIB) regulates myeloid cell maturation by cross-linking with agonistic CD40 mAb in a mouse tumor model, thereby indirectly affecting T cell activation [95]. In some tumor settings, macrophage-secreted IL-10 inhibits IL-12 expression by DC, thereby impairing CD8^+^ T cell-mediated antitumor immunity.

**Figure 3:**
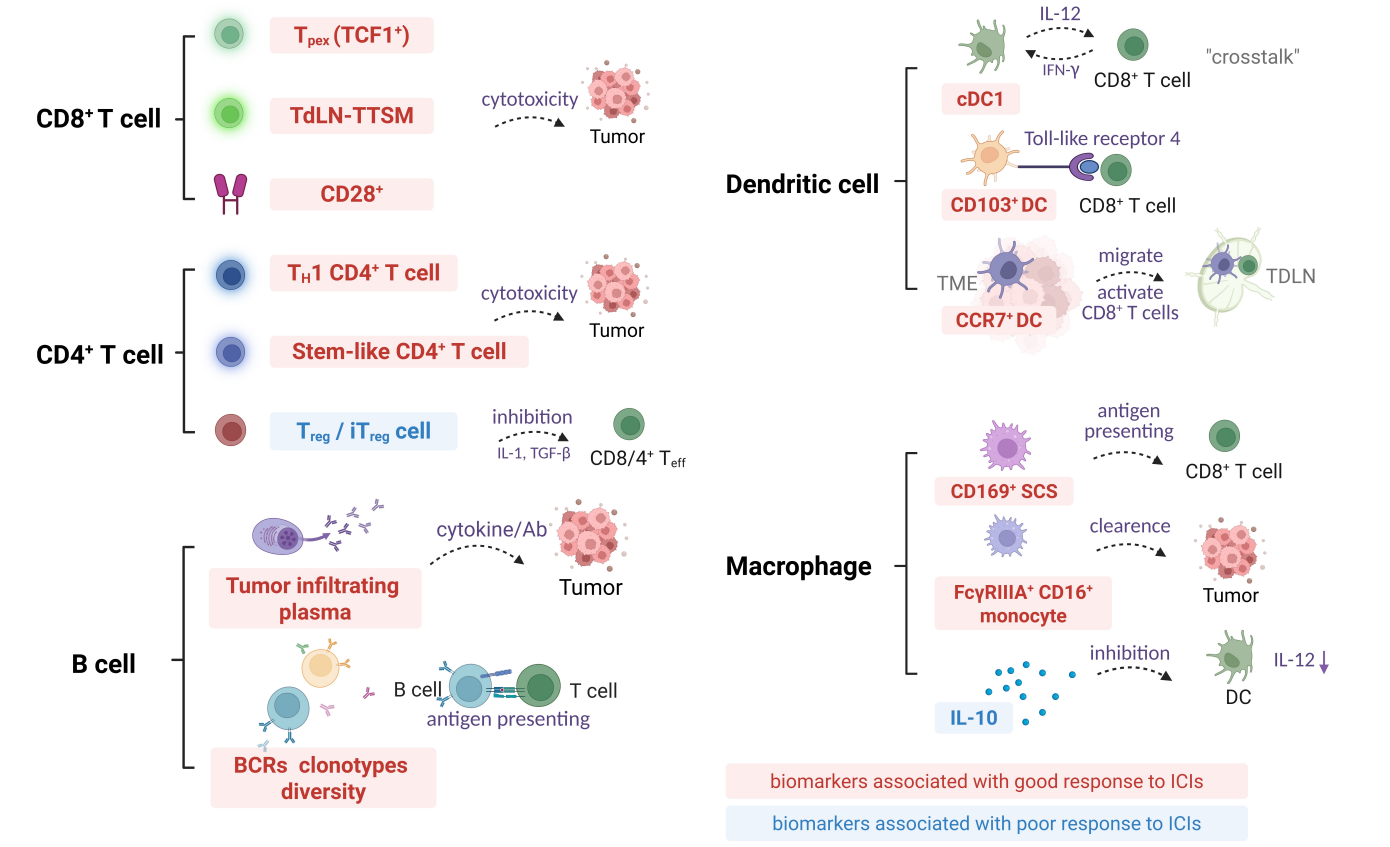
**TDLNs immune cell characteristics as predictive biomarkers for ICI response.** BCRs: B cell receptors; CD8^+^: cluster of differentiation 8^+^; DC: dendritic cell; T_H_1: T helper 1; ICI: immune checkpoint inhibitor; IFN-γ: interferon-γ; IL-12: interleukin-12; iT_reg_: inducible regulatory T cells; SCS: subcapsular sinus; TCF1^+^: T cell factor 1^+^; TDLN: tumor-draining lymph node; TdLN-TTSM: tumor-draining lymph node derived tumor specific memory T cells; T_eff_: effector T cells; TGF-β: transforming growth factor-β; TME: tumor microenvironment; T_pex_: precursor exhausted T cells; T_reg_: regulatory T cells. The downward arrow indicates the downregulation in the molecular expression level. Created in BioRender. Wang, L. (2025) https://BioRender.com/5s504b5

Although the direct correlation between macrophages and ICIs therapy in TDLNs has yet to be thoroughly investigated, available studies suggest that macrophages in TDLNs have the potential to serve as predictive markers of response to ICIs therapy. This dual role of macrophages underscores the need to precisely modulate macrophage function to maximize its role in promoting anti-tumor immune responses when developing new immunotherapeutic strategies. Figure 3 summarizes the immune cell characteristics within TDLNs as predictive features for the response to ICIs.

## TDLNs clinical imaging

Cascone et al. [101] observed that patients who were treated with neoadjuvant ICIs had lymph nodes that were abnormal on imaging but in which pathological evaluation revealed no cancer and instead presented with new noncaseating granulomas—a phenomenon they termed “nodal immune flare (NIF)”. Nodal size and maximum standardized uptake value (SUVmax) before and after treatment were assessed by computed tomography (CT) and 18F-fluorodeoxyglucose positron-emission tomography (PET)-CT analyses. NIF occurred in 16% (7/44) of patients treated with ICIs compared with 0% (0/28) in the neoadjuvant chemotherapy cohort. This suggests that NIF is associated with the inflammatory nodule immune microenvironment, but not with tumor response or treatment-related toxicity. Imaging examination can assess the response of tumor and lymph nodes to treatment non-invasively and early. By measuring the size and SUVmax of lymph nodes, tumor burden, and activity can be quantified to predict treatment response, and it is also of great value for timely adjustment of treatment plans.

## TDLNs metabolism

The Trp-kyrnine-aryl hydrocarbon receptor pathway is involved in the formation of physiological immunosuppression, which includes three enzymes, namely IDO1, tryptophan 2,3-dioxygenase (TDO), and IDO2. IDO1 plays a major role in the formation of immunotherapy resistance. IDO1 is a cytosolic heme-containing enzyme involved in the degradation of tryptophan to kyphoenine [102]. The physiological role of IDO1 is to induce acquired immune tolerance. It can promote the consumption of tryptophan and the accumulation of kynurenine, which has an impact on immune cells: promote the activation of T_reg_, inhibit the activation of T cells, and induce T cell anergic state. IOD1 over-expression in sentinel lymph nodes of melanoma patients reduces the efficacy of anti-CTLA-1-4 and anti-PD1/PD-L1 therapy, and leads to reduced lymphocyte infiltration at the tumor site and poor prognosis [46].

## TDLNs metastasis

Tumor metastasis of lymph nodes refers to the migration of cancer cells from the primary site and colonization in adjacent or distal lymph nodes [103]. It is one of the important characteristics of tumor spread, and has an important impact on treatment decision-making and patient prognosis. Lymph node metastasis is a key indicator in the staging of many solid tumors, such as the Tumor-Node-Metastasis (TNM) system, which usually indicates the aggressiveness of the tumor and the advanced stage of the disease. For instance, in NSCLC lymph nodes metastasis is usually associated with stage III or IV disease, indicating the tumor’s spread from a localized lesion to regional or distant sites. Similarly, in breast cancer, a node-positive status, such as axillary lymph nodes metastasis, is a crucial determinant of adjuvant treatment strategies and correlates with a heightened risk of recurrence [104].

The immune status of lymph nodes is an important factor in predicting the efficacy of ICIs, and lymph nodes with tumor metastasis often show significant immunosuppression, such as immune escape and increased PD-L1 expression, which are associated with worse response to neoadjuvant immunotherapy and shorter disease-free survival. Studies have shown that the number of T_reg_ expressing immunosuppressive coreceptors is increased in TDLNs with metastasis, and these cells are transported to the TME to further suppress tumor-specific immune responses [72]. Early clinical data from neoadjuvant trials suggest that lymph nodes metastasis may limit the response to neoadjuvant ICIs; for example, in resectable NSCLC, the presence of lymph nodes metastasis is associated with worse response to neoadjuvant ICIs therapy and shorter regression free survival [105]. Patients with tumor lymph nodes metastasis before ICIs treatment usually show poor treatment response, but patients with lymph nodes metastasis may have better survival prognosis if they have a major pathological response (MPR) of lymph nodes after ICIs treatment. Deng et al. [106] conducted clinical trial of NSCLC patients receiving neoadjuvant immunotherapy combined with chemotherapy found that a new criteria for MPR of metastatic lymph nodes [mLN^–^ MPR^(+)^] defined by them had good postoperative survival prediction efficiency. Some mLN with pathological responses may present different immune subtypes, which may be related to the prognosis.

## Major challenges to target TDLNs

The immune status and function of TDLNs play a crucial role in the efficacy of ICIs and also serve as rich sources for the identification of novel predictive biomarkers. Mechanistic dissection of the crosstalk between lymph nodes heterogeneous leukocyte populations, as well as stroma cells, is key to the application of lymph nodes as therapeutic markers for ICIs. Exploring the interaction between lymph nodes and TME can enhance our understanding of the role of lymph nodes in the overall immune environment, thereby optimizing immunotherapy strategies. In the previous sections, we demonstrated the theoretical feasibility of utilizing multiple features of lymph nodes as biomarkers for predicting ICIs efficacy, however, it is acknowledged that significant progress is still required before reaching clinical maturity.

### Diversity lymph node research methods

The methods to study lymph nodes include surgical staining to observe tumor metastasis, hematoxylin and eosin (H&E) staining of pathological sections, immunohistochemistry, single-cell sequencing, imaging scanning, metabolomics study, and spatial multi-omics study with tissue morphology preservation. These methods enable us to identify the characteristics of lymph nodes from different perspectives and provide multi-dimensional information for the prediction of ICIs efficacy. Despite these advancements, the approach also has certain limitations. For instance, there are notable discrepancies in consistency between various detection methods and clinical presentations, and conducting a comprehensive analysis of these differences poses a significant challenge. Additionally, single-cell sequencing is not only relatively costly but also involves complex sample processing procedures. Spatial multi-omics analysis similarly faces challenges in data integration and interpretation. In this context, comprehensive analyses of human TDLNs biological samples based on histological or clinical parameters using a variety of methodologies will be of paramount importance.

### Single or sweeping

Retrospective clinical studies of patients with melanoma, thyroid cancer, and breast cancer have found that prophylactic lymph node removal does not improve survival, and the research featured in Xue et al. [107] has shown that surgical removal of TDLNs before tumor implantation and/or anti-PD-1 therapy could reduce treatment efficacy. This supports a central role for TDLNs in the induction of CTL-mediated antitumor cell killing after PD-1 blockade. Therefore, in practice, we prefer to evaluate the status of single lymph nodes to predict the efficacy of ICIs, rather than perform extensive lymph node dissection to reduce the risk of trauma and complications for patients. This selective lymph node evaluation may provide a basis for individualized prediction of the efficacy of ICIs.

### Timing of lymph node detection

In order to predict the efficacy of ICIs, it is important to determine the best timing of lymph node detection, which may include before ICIs treatment, during a specific course of treatment after ICIs treatment, or when sentinel lymph node surgery is performed after immunotherapy. The selection of these timing is of great significance for evaluating the efficacy of ICIs and guiding subsequent treatment strategies. If TDLNs remain in situ prior to ICIs treatment, pre-treatment biopsies or systemic immune population sampling of TDLNs may become important predictive biomarkers. Post-treatment evaluation of resected TDLN may help prognosis or determine whether further adjuvant therapy is needed. Ongoing clinical trials of neoadjuvant ICIs may help define the most effective timing of therapy.

### Lymph node sampling methods

There are two main methods for lymph node detection: non-invasive and invasive. Surgical resection of the whole lymph node or needle biopsy is widely used in clinical practice. Utilizing non-invasive detection techniques for sampling TDLNs together with more clinical information may help us delineate the relationship between TDLNs immune environment and ICIs efficacy. Evaluation of TDLNs in patients with NSCLC by fine-needle aspiration can determine the composition of immune cells [108], which may help predict the response to ICIs.

### Differences among various tumor types

TDLNs of different tumor types exhibit distinct immune characteristics, which implies that predictive markers effective in one tumor type may not be applicable to others. This increases the complexity of research and application, necessitating more in-depth and specific studies for different tumor types.

## Conclusions

In this paper, we systematically reviewed the research progress of TDLNs’ characteristics as potential biomarkers for predicting the efficacy of ICIs. We summarize several key features related to the efficacy of ICIs in TDLNs: 1) dynamic changes in stem-like T cells and T cells: there exists a subset of stem-like CD8^+^ T cells in TDLNs, which, once generated in TDLNs, can proliferate and differentiate independently of sustained antigen stimulation, crucial for long-term anti-tumor immunity. Dynamic changes in CD4^+^ T cells and CD4^+^ T_regs_ are also associated with ICIs efficacy. 2) APC functional status: CCR7^+^ DC subsets migrate from the TME to TDLNs, where they activate and amplify tumor-specific T cells, playing a critical role in the tumor immune cycle. cDC1 cells can cross-present tumor antigens, activate CD8^+^ T cells, and in anti-PD-1 antibody therapy, their secretion of IL-12 stimulates CD8^+^ T cells and enhances the recovery of T cell dysfunction. 3) Additionally, other features of lymph nodes, such as PET-CT imaging features, metabolism, and tumor cell metastasis, have also been shown in studies to be related to ICI treatment response.

We emphasize that ICI efficacy depends on systemic immune activation, a process inadequately captured by single biomarkers. As specialized immunological training centers coordinate immune cell convergence and interactions, the analysis of TDLNs requires systematic evaluation through immune contexture mapping rather than isolated cellular parameter quantification. Instead, a holistic approach should be adopted, considering the composition of immune cells within the lymph nodes, their interactions, and other factors to characterize the immune microenvironment. For instance, employing an immune microenvironment scoring can provide a comprehensive assessment of whether TDLNs are inhibiting or promoting the response to ICIs. Moreover, integrating the characteristics of TDLNs with well-established predictive markers such as PD-L1 expression, TMB, and MSI is anticipated to enhance the accuracy of predicting treatment efficacy. Advanced techniques including spatial multi-omics and imaging evaluations are reshaping our understanding of the immunoregulatory mechanisms of TDLNs. These methodologies hold significant potential for elucidating the mechanism of TDLNs in tumor immunity and are expected to provide a robust scientific foundation for subsequent mechanism research and clinical applications.

The standardized validation of TDLNs biomarkers across diverse tumor types and demographic cohorts, coupled with the establishment of optimal temporal window for biomarker detection, and the development of multiplexed biomarkers protocol, constitutes critical research priorities for establishing TDLNs as reliable predictive biomarkers for ICIs. Through systematic refinement of analytical methodologies and integration of multidimensional predictive frameworks, TDLNs are expected to play a crucial role in enhancing the efficacy of ICIs and guiding personalized immunotherapy.

## Abbreviations

APCs: antigen-presenting cells
BCRs: B cell receptors
CD279: cluster of differentiation 279
CT: computed tomography
CTLA-4: cytotoxic T-lymphocyte associated protein 4
CTLs: cytotoxic T-lymphocytes
DCs: dendritic cells
HNSCC: head and neck squamous cell carcinoma
ICIs: immune checkpoint inhibitors
IDO1: indoleamine 2,3-dioxygenase 1
IFN-γ: interferon-γ
IL-7: interleukin-7
MHC-I: major histocompatibility complex class I
mLN: metastatic lymph nodes
MPR: major pathological response
MSI: microsatellite instability
NIF: nodal immune flare
NK: natural killer
NSCLC: non-small cell lung cancer
PD-1: programmed death protein 1
PD-L1: programmed death ligand 1
PET: positron-emission tomography
RCC: renal cell carcinoma
SCS: subcapsular sinus
SUVmax: maximum standardized uptake value
TAAs: tumor-associated antigens
TCF1: T cell factor 1
TCR: T cell receptor
TDLNs: tumor-draining lymph nodes
TdLN-TTSM: tumor-draining lymph node derived tumor specific memory T cells
T_eff_: effector T cells
T_ex-int_: transitional exhausted T cells
T_ex-term_: terminally exhausted T cells
TGF-β: transforming growth factor-β
T_H_: T helper
TLR9: Toll-like receptor 9
TLSs: tertiary lymphoid structures
TMB: tumor mutational burden
TME: tumor microenvironment
T_mem_: memory T cells
T_naive_: naive T cells
TNBC: triple negative breast cancer
TNF-α: tumor necrosis factor-α
T_pex_: precursor exhausted T cells
T_reg_: regulatory T cells
TSAs: tumor-specific antigens
uiLNs: uninvolved regional lymph nodes

## Declarations

### Copyright

© The Author(s) 2025.
